# Supplementary material for: Do Mass Spectrometry-Derived Metabolomics Improve the Prediction of Pregnancy-Related Disorders? Findings from a UK Birth Cohort with Independent Validation
Source: Metabolites. 2021 Aug 10;11(8):530. doi: 10.3390/metabo11080530 (PMC8399752; doi:10.3390/metabo11080530)
Supplement: Supplementary file 1 [file metabolites-11-00530-s001.zip › metabolites-1282529-SI/Supplementary_figures.pdf]

Supplementary figures

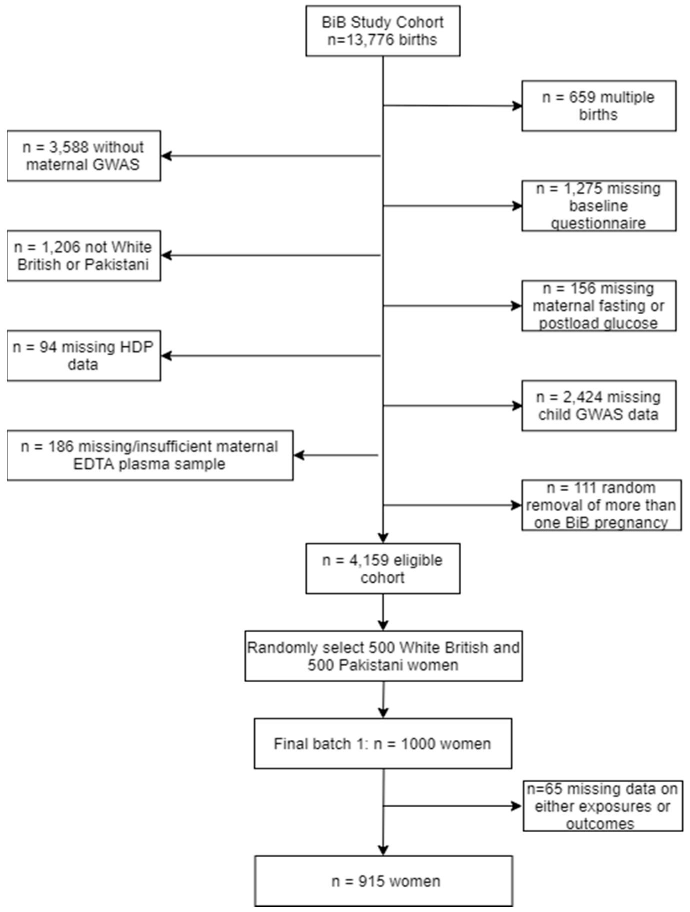

Figure S1a: Born in Bradford 1,000 flow of participants sample selection

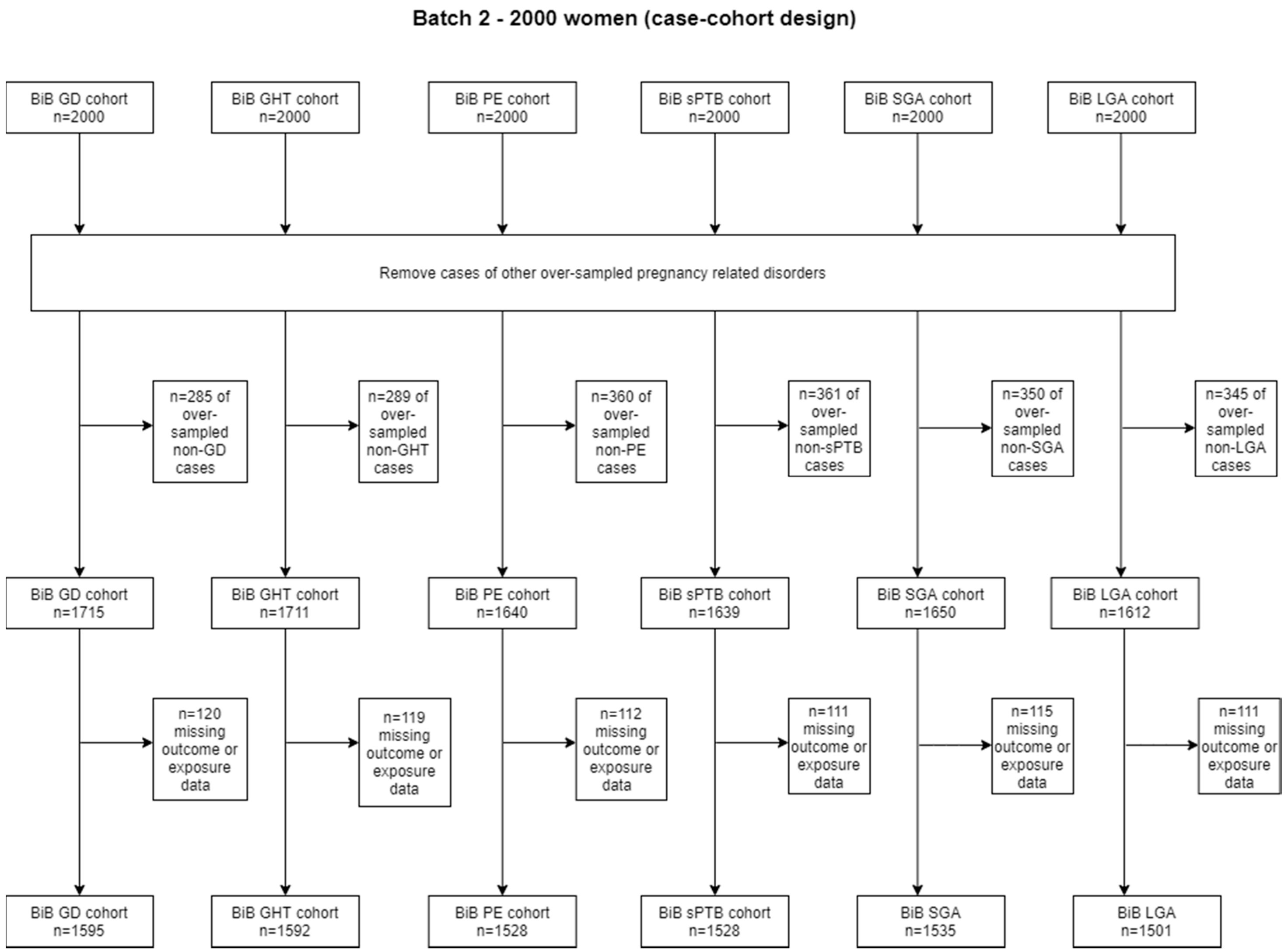

Figure S1b: Born in Bradford 2,000 flow of participants sample selection

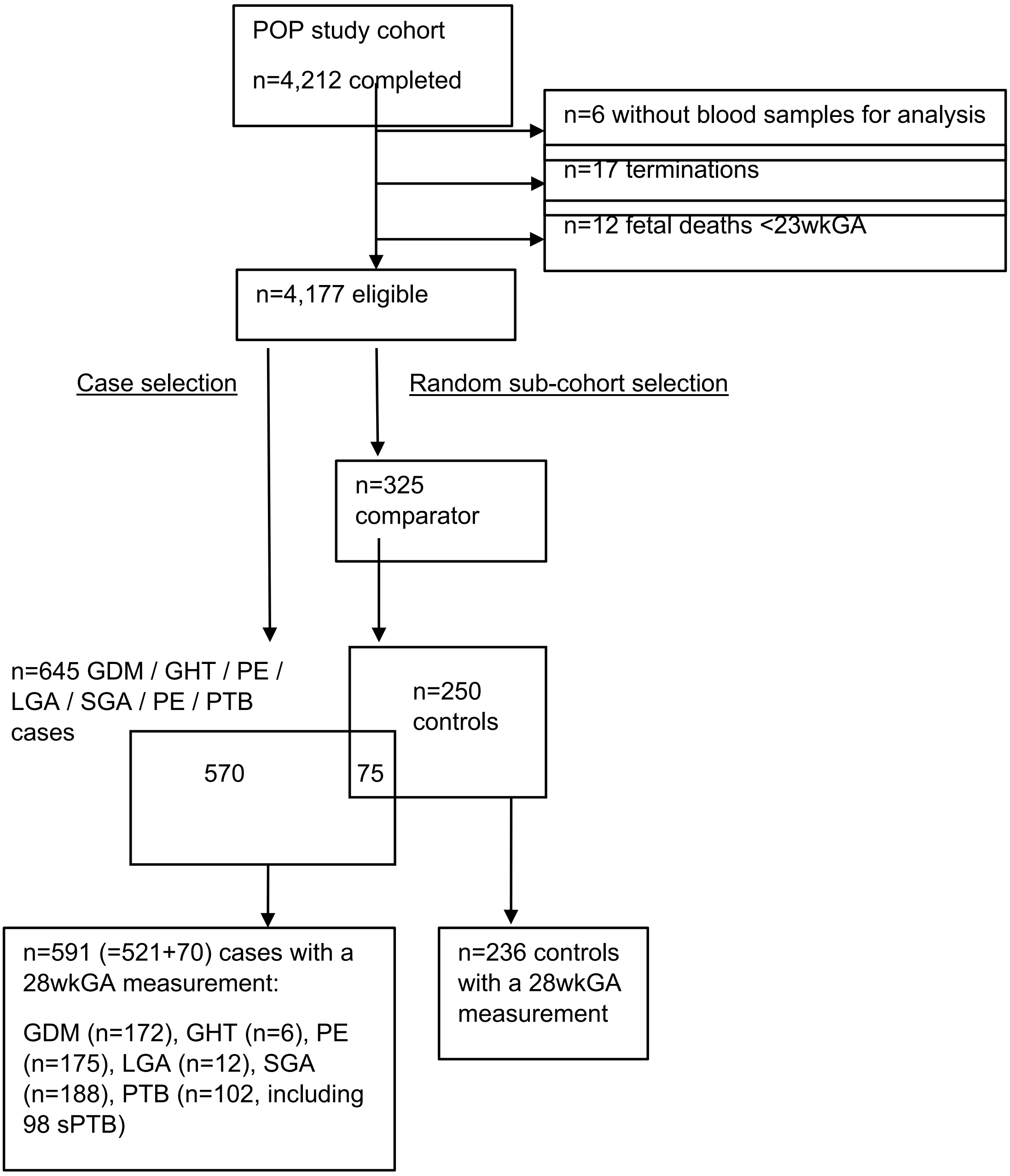

Figure S1c: Pregnancy Outcome Prediction study sample selection.

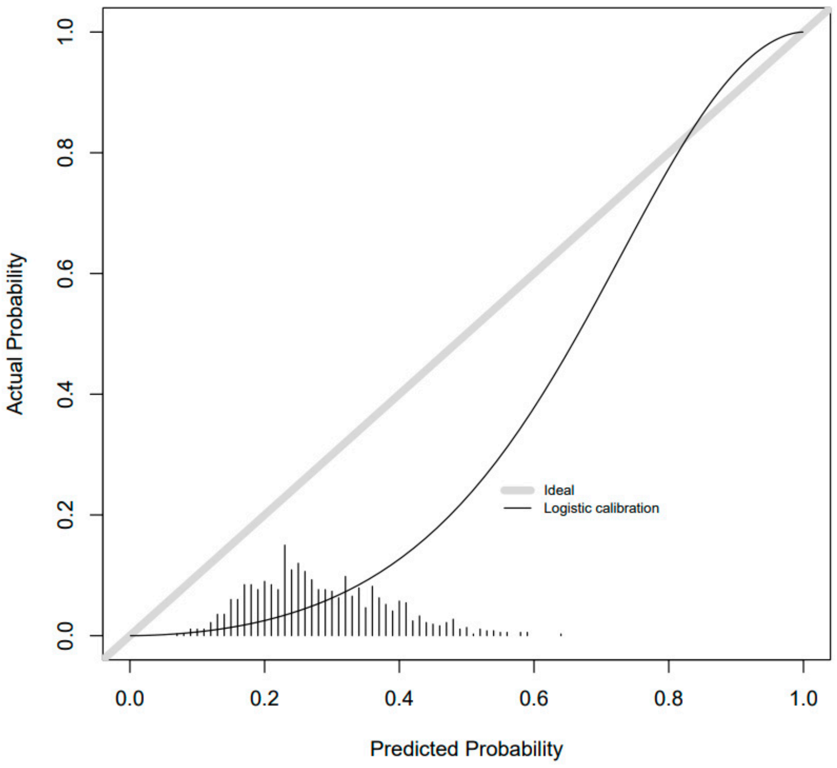

Figure S2: The calibration slope for the combined model of gestational hypertension prediction in the Born in Bradford 1000 cohort

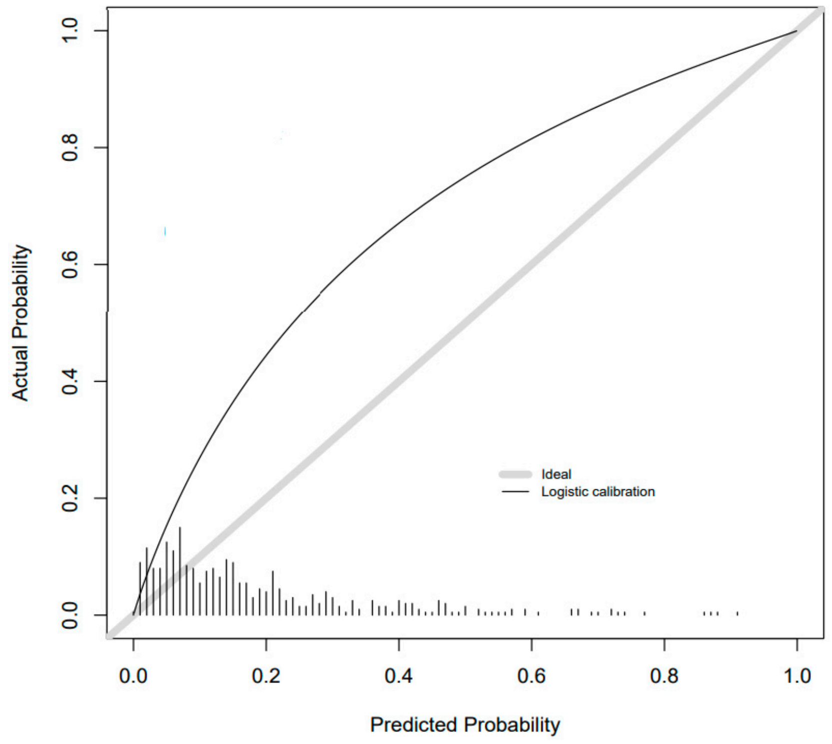

Figure S3: The calibration slope for the combined model of gestational diabetes prediction in the Pregnancy Outcome Prediction study

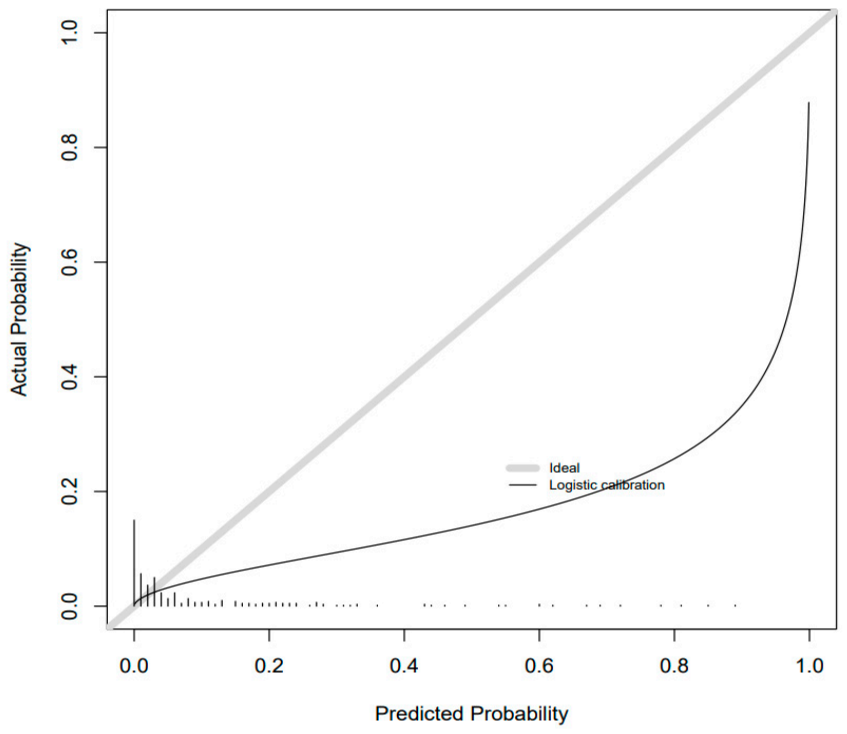

Figure S4: The calibration slope for the combined model of large for gestational age prediction in the Pregnancy Outcome Prediction study

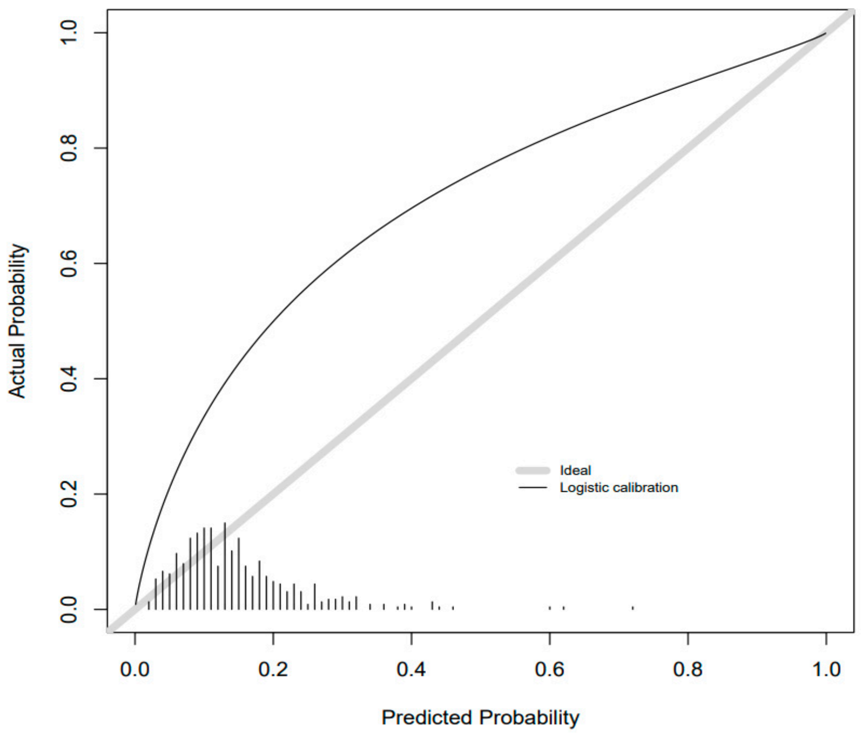

Figure S5: The calibration slope for the combined model of small for gestational age prediction in the Pregnancy Outcome Prediction study

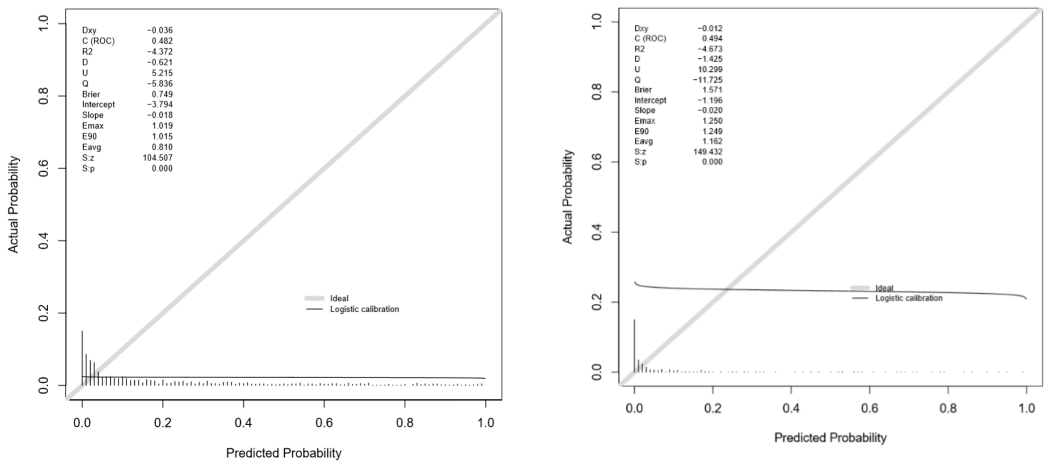

Figure S6: The calibration slope for the combined model of spontaneous preterm birth prediction in the Born in Bradford 1000 cohort and the Pregnancy Outcome Prediction study

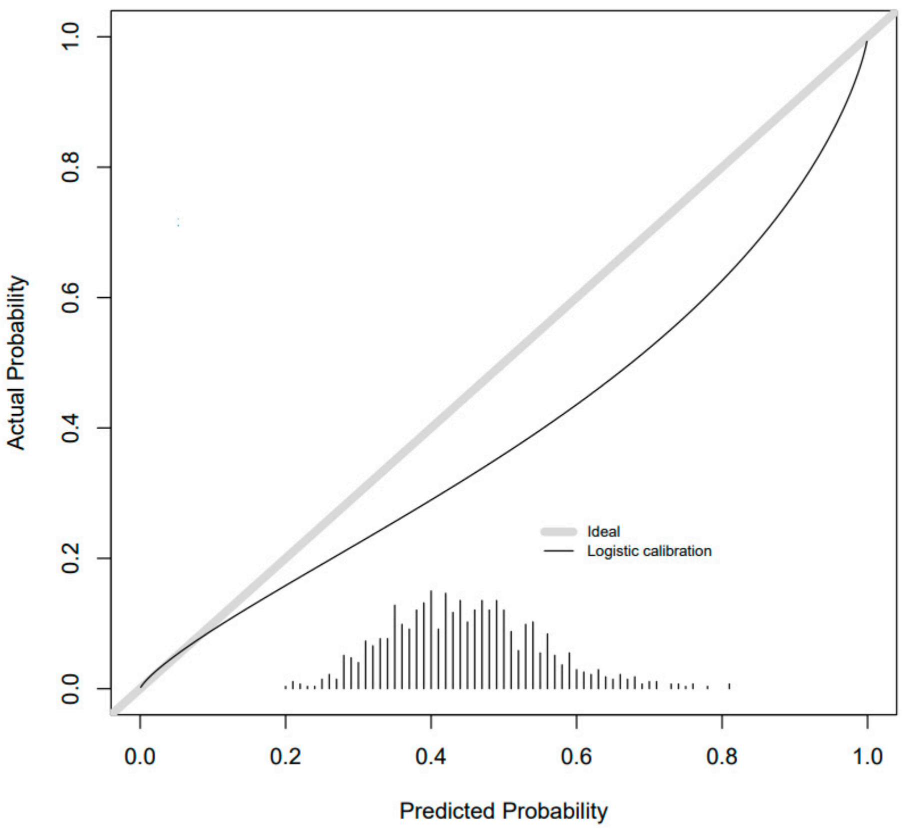

Figure S7: The calibration slope for the combined model of all pregnancy related disorder prediction in the Born in Bradford 1000 cohort
